# Reviewing perianal abscess management and recurrence: lessons from a trainee perspective

**DOI:** 10.1111/ans.17750

**Published:** 2022-04-29

**Authors:** Mina Sarofim, Kevin Ooi

**Affiliations:** ^1^ Department of Colorectal Surgery Bankstown Hospital Sydney Australia; ^2^ School of Medicine University of New South Wales Sydney Australia

**Keywords:** perianal abscess, training

## Abstract

**Background:**

Perianal abscesses are a common surgical emergency. Due to their perceived ease, drainage is often delegated to junior trainees with varying levels of experience. The purpose of this study is to evaluate the current trend in perianal abscesses management at our institution, and identify factors that predict subsequent fistula formation or abscess recurrence.

**Methods:**

All acute patients admitted to a major teaching hospital who required surgical drainage of a perianal abscess were analysed over a two‐year period from January 2019 to December 2020. Patient demographics, clinical and laboratory findings were retrospectively reviewed. Proceduralist experience, operative management strategy and recurrence rates (fistula or abscess) were analysed.

**Results:**

The mean age of patients was 43 years old, and 73% were male. Trainees performed 96% of the procedures. Re‐presentation with a fistula or abscess recurrence requiring further surgery was 31%. Comorbidities of IBD, diabetes, or malignancy were present in one‐third of patients and significantly increased the risk of recurrence (*P* = 0.01). Searching for a fistula tract was performed in 41% of cases but did not reduce recurrence (*P* = 0.9). Seton insertion occurred in 10%, and fistulotomy in 2%.

**Conclusion:**

Perianal abscess drainage at our institution is almost exclusively performed by trainees, the majority of which occurs after‐hours. Patients who present with a fever, inflammatory bowel disease, diabetes mellitus or malignancy are at an increased risk of recurrent abscess or a subsequent fistula after drainage, and input from an experienced surgeon may be of value when considering seton insertion or fistulotomy.

## Abstract 2

Perianal abscess management is primarily done by trainees of varying experience. This retrospective review evaluates current trends at our institution and identify factors that predict abscess recurrence.

## Introduction

Perianal abscesses are a daily presentation to emergency departments. They are most commonly assessed and drained by surgical trainees of varying experience. The underlying pathophysiology is well taught in surgical texts; they arise due to obstruction of the anal glands which traverse the internal sphincter and open at the dentate line.^1^ More accurately they are termed anorectal abscesses, classified anatomically as perianal, ischiorectal, intershpincteric or supralevator.

Definitive management is perhaps mistakenly perceived as a ‘low acuity’ or ‘simple’ incision and drainage of sepsis, often delegated to the junior trainee or performed after‐hours. While this approach may apply to abscesses elsewhere on the body, the intricate sphincter anatomy can be deceiving as evidenced by the fact that one‐third will re‐present with fistula in ano.^2^ Intra‐operative decision making may also be complex: searching for a fistula tract, seton insertion, fistulotomy or performing wound cultures remain contentious management strategies and experienced surgeons can provide invaluable input.^3^

The purpose of this study therefore is to evaluate the current trend of perianal abscess management at our institution, and identify clinical or operative factors which can assist trainees reduce subsequent fistula formation or abscess recurrence.

## Methods

All acute patients admitted to a major teaching hospital who required surgical drainage of a perianal abscess were analysed over a 2‐year period from January 2019 to December 2020. Patient demographics, clinical and laboratory findings were retrospectively reviewed. Proceduralist experience and operative strategy were documented. Searching for a fistula tract was defined as using an anal retractor and gently passing a Lockhart‐Mummery probe or instillation of hydrogen peroxide and identifying an internal opening. A recurrence was defined as a previous or subsequent presentation with a perianal abscess requiring surgery, or a subsequent examination under anaesthesia which identified a fistula tract. Statistical analysis was performed using Chi‐squared tests with a significance value *P* = 0.05.

HREC approval was obtained for this project – low/negligible risk pathway (Ref 2022/ETH00214). The data that support the findings of this study are available from the corresponding author upon reasonable request.

## Results

A total of 78 patients required perianal abscess drainage during the 2‐year period. The mean age was 42.9 years and 73% were male. Trainees performed 96% of the procedures. Wound swab for culture was performed in 77%. Abscess recurrence rate was 31%, and these surgical drainage procedures were also all performed by trainees.

Comorbidities of inflammatory bowel disease (IBD), diabetes, or malignancy were present in one‐third of patients and significantly increased the risk of subsequent fistula formation or abscess recurrence (*P* = 0.01). Interestingly, a documented fever (T > 38°) in the emergency department was present in 17%, and these patients had a significantly higher risk of recurrence (*P* = 0.04). Gender, symptom duration or elevated inflammatory markers did not significantly affect recurrence rate.

After‐hours surgery was performed in 58% of cases. Searching for a fistula tract (using an anal retractor, Lockhart‐Mummery probe or instillation of hydrogen peroxide) was performed in 41% of cases but did not reduce recurrence (*P* = 0.9). Seton insertion occurred in 10%, and fistulotomy in 2%. Management using a seton or fistulotomy was performed at similar rates both in‐hours and after‐hours, 15% compared with 11% respectively.

## Discussion

Trainees perform most perianal abscess drainages which may be complex procedures due to the anatomy involved, but this practice appears safe as overall recurrence rates are in keeping with current literature.^2^ Not surprisingly, recurrence or fistulae are higher in patients with comorbidities associated with bowel pathology such as IBD, or immunosuppression such as malignancy or diabetes. However, a novel finding in our series was the predictive value of fever on arrival to hospital, which has not been previously described. Patients identified at high risk pre‐operatively may benefit from input from an experienced surgeon, as evidence suggests this increases identification and definitive management of underlying fistula tracts.^3^

From a practical viewpoint, skin incisions close to the anal verge ensure that any potential fistula will have a short length. Any search for a fistula tract, using anal retractors and Lockhart‐Mummery probes must be performed carefully to avoid creating false tracts. Wound packing is often up to the discretion of the proceduralist and the cavity size; however, randomized trials have demonstrated no significant benefit other than increasing post‐operative pain.^4^, ^5^

Performing a fistulotomy or seton insertion at the time of drainage remains a controversial strategy. A number of trials have reported conflicting outcomes, and the traditional teaching is that delaying fistula management is preferred, as the acute inflammation and oedematous tissue have resolved. The advertised benefits of faster wound healing, decreased abscess recurrence and fistula formation must be balanced against the three‐fold increased risk of incontinence^6^, ^7^ and therefore considered on a case‐by‐case basis after experienced surgeon consultation.

Wound cultures are not considered routine when draining perianal abscesses, although they can differentiate patients into those with bacteria arising from the colon versus the skin. The former suggests an abscess of cryptoglandular origin which traditionally was thought to increase the risk of recurrence or fistula formation; however, this has not been consistently demonstrated.^8^

Surgical examination under anaesthesia is traditionally considered the gold standard for defining an abscess or fistula tract. The use of imaging in select patients with suspected complex pathology however can provide an anatomical road map to avoid sphincter damage or creation of false tracts. Readily available and rapid computed tomography differentiates an abscess from severe cellulitis, or identifies extension into the supralevator space; the limitation being its ability to identify fistula tracts as they share the same appearance as inflammatory stranding.^9^, ^10^ Magnetic resonance imaging avoids radiation exposure and provides excellent tissue resolution for complex fistulae patients in an elective setting or underlying IBD, however, is rarely available in the emergency department for a patient on the brink of sepsis. Similarly, due to the availability and expertise required, endoscopic ultrasound is an unrealistic option that would be difficult to tolerate by patients with a presenting complaint of significant perianal pain.^9^

A limitation to this present study is the undetermined effect on anal sphincter function and incontinence post‐surgery. Short‐ and long‐term function following perianal abscess drainage presents itself as a topic of future research to ensure trainees are producing acceptable results.

## Conclusion

Perianal abscess drainage at our institution is almost exclusively performed by trainees, the majority of which occurs after‐hours. This practice appears to successfully manage sepsis and produces comparable recurrence rates to the known literature. Trainees would do well to recognize patients who present with a fever, IBD, diabetes mellitus or malignancy as they significantly predict a risk of recurrent abscess or a subsequent fistula after drainage. Input from an experienced surgeon or pre‐operative imaging is a good strategy for the stable patient identified at high risk of hiding complex pathology. In addition to simple drainage, searching for a fistula tract, seton insertion or fistulotomy did not significantly reduce recurrence in our series, and since our study has not assessed continence after treatment this should not be routinely done by surgical trainees.

## Conflict of interest

None declared.

## Aurhor contributions

**Mina Sarofim:** Conceptualization; data curation; formal analysis; investigation; writing – original draft; writing – review and editing. **Kevin Ooi:** Conceptualization; project administration; supervision; writing – review and editing.
